# Cancer risks in populations living near landfill sites in Great Britain

**DOI:** 10.1038/sj.bjc.6600311

**Published:** 2002-06-05

**Authors:** L Jarup, D Briggs, C de Hoogh, S Morris, C Hurt, A Lewin, I Maitland, S Richardson, J Wakefield, P Elliott

**Affiliations:** The Small Area Health Statistics Unit (SAHSU), Department of Epidemiology and Public Health, Imperial College, St Mary's Campus, Norfolk Place, London W2 1PG, UK

**Keywords:** landfills, bladder cancer, brain cancer, hepatobiliary cancer, leukaemia

## Abstract

Previous studies have raised concerns about possible excess risks of bladder, brain and hepatobiliary cancers and leukaemias near landfill sites. Several cancers have been implicated, but no consistent pattern has emerged. We present a large nationwide analysis of selected cancers near landfill sites in Great Britain. The base population comprised people living within 2 km of 9565 (from a total of 19 196) landfill sites that were operational at some time from 1982 to 1997, with populations living more than 2 km from a landfill as reference. Risks of cancers at the above sites were computed with adjustment for age, sex, year of diagnosis, region and deprivation. National post-coded registers provided a total of 341 856 640 person–years for the adult cancer analyses and 113 631 443 person–years for childhood leukaemia. There were 89 786 cases of bladder cancer, 36 802 cases of brain cancer, 21 773 cases of hepatobiliary cancer, 37 812 cases of adult leukaemia and 3973 cases of childhood leukaemia. In spite of the very large scale of this national study, we found no excess risks of cancers of the bladder and brain, hepatobiliary cancer or leukaemia, in populations living within 2 km of landfill sites. The results were similar if the analysis were restricted to landfill sites licensed to carry special (hazardous) waste. Our results do not support suggestions of excess risks of cancer associated with landfill sites reported in other studies.

*British Journal of Cancer* (2002) **86**, 1732–1736. doi:10.1038/sj.bjc.6600311 www.bjcancer.com

© 2002 Cancer Research UK

## 

Several studies have suggested associations between residence near landfills containing hazardous waste and cancer. The major studies are listed in Table 1

**Table 1:**
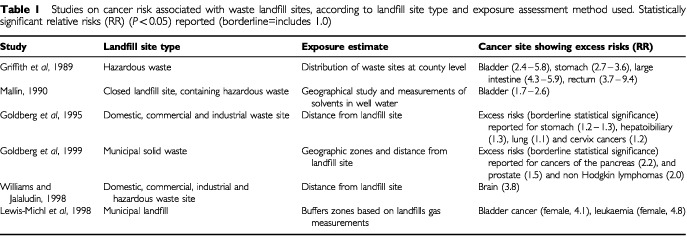
Studies on cancer risk associated with waste landfill sites, according to landfill site type and exposure assessment method used. Statistically significant relative risks (RR) (*P*<0.05) reported (borderline=includes 1.0)

, showing that bladder cancer is the most frequently reported malignancy associated with landfills. The detoxifying properties of the liver suggest that environmental toxicants may accumulate in the liver and biliary tract; small excess risks of liver cancer were reported in previous studies around UK incinerators (Elliott *et al*, 1996, 2000). Several studies have shown an association between brain cancer and exposure to pesticides (Bohnen and Kurland, 1995), which are frequently used on landfill sites. Leukaemia has been associated with exposure to volatile organic compounds (VOCs), such as benzene (IARC, 1987b), which occur in emissions from landfill sites. Although several other cancers have been implicated, no consistent pattern has emerged (Vrijheid, 2000).

A wide range of waste degradation products may be released into the environment from landfill sites. Gaseous releases include primarily methane and carbon dioxide as well as smaller quantities of hydrogen sulphide, VOCs and metal vapours (Zmirou *et al*, 1994; Hamar *et al*, 1996; Ward *et al*, 1996). Several of these compounds (such as benzene (IARC, 1987b) and cadmium (IARC, 1993)) are classified as carcinogenic to humans (Group 1) by the International Agency for Research on Cancer (IARC). Others are considered probably (Group 2A; e.g. formaldehyde (IARC, 1995)) or possibly (Group 2B; e.g. styrene (IARC, 1994) and lead (IARC, 1987a)) carcinogenic to humans. Leaching and runoff of waste decomposition products may occur (El-Fadel *et al*, 1997), not only while the site is being operated, but also after closure, as waste products continue to decay (Bozkurt *et al*, 2000). Human exposure to these releases potentially occurs via inhalation of polluted air, ingestion of contaminated water, or skin contact with contaminated water or soil. Monitoring of pollutants around landfill sites indicates that detectable levels of pollution tend to be confined to the immediate proximity of the site (United States Environmental Protection Agency, 1999). A recent WHO report suggested that any potential exposure is likely to be limited to 1 km from landfill sites by the air pathway, and 2 km by the water pathway (WHO, 2000). The aim of the present study was to examine the incidence of bladder, brain and hepatobiliary cancers as well as childhood and adult leukaemia near landfills in Great Britain.

## MATERIALS AND METHODS

### Landfill sites

Databases on landfill sites were compiled in a geographical information system (GIS), based on core data for England and Wales, provided by the Environment Agency, and for Scotland, provided by the Scottish Environment Protection Agency. In both cases, different data sets, compiled at different times, had to be merged to produce a comprehensive and consistent listing of all known landfill sites. The resulting data set comprised 19 196 landfill sites (17 746 in England and Wales and 1450 in Scotland) (Briggs *et al*, 2001).

### Cancer data

We used data from England, Wales and Scotland postcoded registers, held by the UK Small Area Health Statistics Unit (SAHSU). The cancer incidence registers (Office for National Statistics (ONS) for England and Wales, Information and Statistics Division (ISD) for Scotland) included data from 1983–1997, except for Wales where data from 1983–1994 were available. For the denominators, data at enumeration district (ED) level from the 1981 and 1991 censuses were used. Populations for the years 1983 to 1990 were calculated by interpolation (Arnold, 1999, pp 10–24), and between 1992 and 1997 by distributing the ONS mid year district level estimates to EDs according to the proportions found in the 1991 census. These ED level population estimates were then used to calculate postcode level populations by point in polygon methods weighted by number of households in each postcode.

Cases were coded to the International Classification of Diseases (ICD) version 9 from 1983 to 1994, and to version 10 thereafter. Primary outcomes were all leukaemia (ICD9 204–208, ICD10 C91–C93) in children aged 0–14 years and in adults (15+ years); bladder cancer (ICD9 188, 236.7, ICD10 C67, D41.4); brain cancer (ICD9 191–192, 225, 237.5, 237.6, 237.9, ICD10 C70–C72, D32, D33, D43), and hepatobiliary cancer (ICD9 155–156, ICD10 C22–C24).

### Data preparation

For the large majority of landfill sites the only locational data available were point co-ordinates (usually of the gateway). A range of locational checks was carried out on these data (e.g. by intersecting site co-ordinates with other, independent locational data, by comparison of co-ordinates given in different data files, and by intersection with district and county boundaries) and these showed that they were also subject to considerable error in some cases. This was confirmed by field visits to a selection of sites, using global positioning systems (GPS), which showed errors of 200–500 metres, although with larger errors for a small minority of sites. The data providers corrected locational errors, where possible, but despite this site co-ordinates must be seen as only a poor approximation of the location and extent of sites that may be several tens (and in some cases several hundreds) of hectares in area and may change markedly in extent over time. Similar problems also occur with the postcode data, used to define place of residence. Although these are notionally accurate to a few metres as point locations, they again represent areas of several tens of metres (in densely populated urban areas) to >1000 square metres (in rural areas). They are also subject to recording errors (sometimes of several hundred metres) and change over time in response to changing postal delivery patterns. In addition, landfill sites are highly clustered, so that individual postcodes may lie close to 30 or more different sites. For all these reasons, it was not considered meaningful to construct sophisticated measures of putative exposure to landfill sites. As a proxy for exposure, we therefore constructed buffer zones around each of the 19 196 landfill sites, using GIS techniques. In view of the limited locational accuracy of the data, a 2 km buffer zone was used: finer subdivision of distance from landfill sites (e.g. by constructing 1 km buffer zones) was not considered meaningful (Elliott *et al*, 2001a). The 2 km resolution used in this study was similar to or higher than that of previous studies (Dolk *et al*, 1998; Fielder *et al*, 2000) and at the likely limit of dispersion for landfill emissions to include both air and water pathways, and possible dispersion by birds or animals (WHO, 2000; Elliott *et al*, 2001a).

These 2 km zones were then intersected with the ca. 1.6 million postcodes in Great Britain and the exposure status of each postcode within the buffer zones classified year-on-year according to the operational status (before opening, operating, closed) and waste type (special, non-special, unknown) of the associated landfill sites, using a rule-based approach (Briggs *et al*, 2001). The study base comprised landfill sites that were operational at some time between 1982 and 1997; therefore postcodes for 9631 sites (49%), which closed before 1982, opened after 1997, or for which operational data were missing or incomplete, were excluded. This left 9565 sites, comprising 774 special (hazardous) sites, 7803 non-special sites and 988 handling ‘unknown’ waste types. Postcodes lying outside the 2 km buffer zones of all landfill sites, in all years, were classified to the reference area.

Postcoded health and denominator data were matched to landfill sites by intersecting the buffer zones around the landfill sites with postcode centroids. We ‘lagged’ exposure by 1 year (childhood leukaemia) or 5 years (all other cancer outcomes) to allow for relevant latency periods; thus we examined data for the adult cancers from 1987–1997 and for childhood leukaemia from 1983–1997. Postcodes were assigned to tertiles of the national distribution of Carstairs' scores, an index of deprivation based on 1991 census statistics. (Carstairs and Morris, 1989), derived at the Enumeration District level.

More detailed descriptions of these methods have been published elsewhere (Briggs *et al*, 2001; Elliott *et al*, 2001a,b).

### Statistical analysis

Risks for the exposed population relative to the reference population were calculated using indirect standardisation; standard stratification was felt to be too unstable because of the relatively small population falling in the reference group. Thus, we constructed a Poisson model for the observed reference data with a regression function of the covariates of interest; age, sex, year of diagnosis and standard region (*n*=10). The most appropriate model for each cancer outcome was chosen by ascending stepwise selection. The selection procedure was repeated without adjustment for deprivation, and the two models constrained (where necessary) to differ only in terms of deprivation. Model predictions were then used as the reference rates in calculating expected numbers.

In order to assess the sensitivity of the results to these models, we also examined results from an alternative model that included, in addition, the most significant term excluded at the last step. Further models were run including a measure of urban/rural status.

We calculated 99% confidence intervals around the relative risk estimates using a Poisson model for rare events, assuming a common relative risk for all landfill sites. To the extent that the model assumptions fail to hold (for example, because of data anomalies, unmeasured confounding or sampling variability in the rates) some degree of over-dispersion and a widening of the confidence intervals is to be expected.

## RESULTS

Overall, 341 856 640 person–years for the adult cancers and 113 631 443 person–years for childhood leukaemia were included in the study. Within 2 km of landfills, 31, 34 and 35% of the population were in Carstairs' tertiles 1 (most affluent), 2 and 3 (most deprived) respectively compared with 44, 32 and 23% respectively in the reference area. Thus, the populations living near landfills were more deprived than populations in the reference area.

Table 2

**Table 2:**
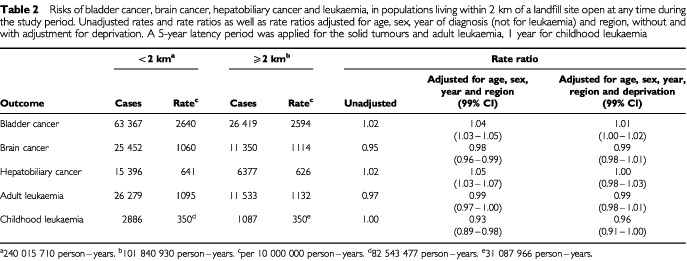
Risks of bladder cancer, brain cancer, hepatobiliary cancer and leukaemia, in populations living within 2 km of a landfill site open at any time during the study period. Unadjusted rates and rate ratios as well as rate ratios adjusted for age, sex, year of diagnosis (not for leukaemia) and region, without and with adjustment for deprivation. A 5-year latency period was applied for the solid tumours and adult leukaemia, 1 year for childhood leukaemia

shows the risks of bladder cancer, brain cancer, hepatobiliary cancer and leukaemia within 2 km of all sites open at any time during the study period. A 4% excess of bladder cancer in the models with deprivation excluded reduced to 1% (99% confidence limits: 0–2%) once deprivation was added. With full adjustment, no excess risk of any other cancer was found, nor was there excess risk of any cancers near the sub-set of landfill sites licensed to carry special (hazardous) waste (Table 3

**Table 3:**
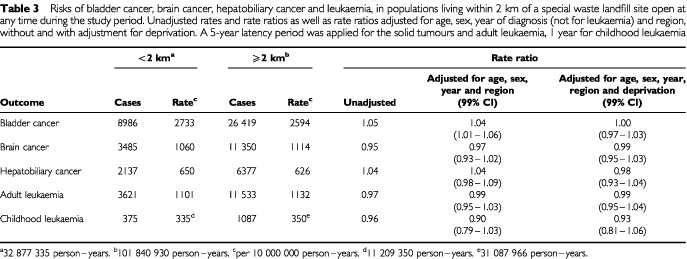
Risks of bladder cancer, brain cancer, hepatobiliary cancer and leukaemia, in populations living within 2 km of a special waste landfill site open at any time during the study period. Unadjusted rates and rate ratios as well as rate ratios adjusted for age, sex, year of diagnosis (not for leukaemia) and region, without and with adjustment for deprivation. A 5-year latency period was applied for the solid tumours and adult leukaemia, 1 year for childhood leukaemia

). The results were robust to the models used in the sensitivity analysis. Detailed results are available in a downloadable report at the Department of Health web-site (Elliott *et al*, 2001b).

## DISCUSSION

This is by far the largest study to report on the possible association between residence near landfill and cancer risk. We did not find any excess risks for the cancers studied, in contrast to previous studies where excess risks of bladder cancer (Griffith *et al*, 1989; Lewis-Michl *et al*, 1998; Mallin, 1990), brain cancer (Williams and Jalaludin, 1998), hepatobiliary cancer (Goldberg *et al*, 1995) and leukaemia (Lewis-Michl *et al*, 1998) have been reported.

In interpreting our results, the possibility of a false negative finding needs to be considered. Various sources of error and uncertainty are present in the data. The landfills data, for example, are subject to errors in location, operating dates and classification of waste types. Although every effort was made to obtain as complete as possible a national inventory of waste sites, nonetheless some landfill sites may be missing from the database (especially older sites which closed before licensing was enforced). Use of a point location (the gateway or centroid of the boundary polygon) to define sites contributes to these uncertainties: landfill sites vary greatly in terms of their surface area, from 50 m^2^ to 70 million m^2^ (average 64 600 m^2^), and the areas and locations change over time as sites evolve. Postcode locations (used to locate the cancer cases with respect to landfill sites) are only accurate to around 100 metres on average.

These uncertainties precluded the use of more sophisticated measures to define exposure near a landfill, and no direct measures were available. There is in any case considerable uncertainty as to the extent of any possible exposure to chemicals found in landfill sites (Pershagen, 1998). Such field monitoring as has been undertaken suggests that pollutants released from landfill sites are detectable only within very small distances of the sites (United States Environmental Protection Agency, 1999). It is therefore possible that any very local effects near landfill sites within our study, or perhaps effects restricted to a small sub-set of landfill sites only, may not have been detected. Possible effects of multiple, or differential, exposures from different landfill sites were also not considered. In addition, latency times, in particular for the solid tumours, and migration in and out of the study areas, may give rise to substantial misclassification with respect to potential exposure to landfill, leading to dilution of any potential effect. However, given that 80% of the population lives within 2 km of a landfill site and is therefore considered as ‘exposed’ in this study, it is likely that a person who moves out of an ‘exposed’ area will move into another ‘exposed’ area. Thus, the potential bias of migration may be less pronounced in this study than in other epidemiological studies where potential exposure commonly is confined to a small part of the population.

In an ecological study such as that considered here, the sources of bias are more complicated than in an individual-level study, and hence the interpretation of estimated rates is more complex (Greenland and Robins, 1994). In particular, the within-area variability in exposure and potential confounders leads to a greater possibility of confounding, and in the absence of within-area data, it may not be possible to control adequately for such confounding. In this particular example, no effect of residence near landfill sites on cancer risk was found, although it is of course possible that unmeasured confounding working in the opposite direction could have masked a true effect.

In conclusion, we found no excess risks of bladder, brain or hepatobiliary cancer, or leukaemia, in populations living close to landfill sites. Our results do not support suggestions of excess risks of cancer associated with landfill sites reported in other studies.
